# Insights on CXC chemokine receptor 2 in breast cancer: An emerging target for oncotherapy

**DOI:** 10.3892/ol.2019.10957

**Published:** 2019-10-03

**Authors:** Fengzhu Guo, Lang Long, Jiantao Wang, Yuyi Wang, Yanyang Liu, Li Wang, Feng Luo

**Affiliations:** Lung Cancer Center, Cancer Center, State Key Laboratory of Biotherapy, West China Hospital of Sichuan University, Chengdu, Sichuan 610041, P.R. China

**Keywords:** breast cancer, CXC chemokine receptor 2, interleukin-8, molecular mechanism, targeted therapy

## Abstract

Breast cancer is the most common malignant neoplasm in women worldwide, and the treatment regimens currently available are far from optimal. Targeted therapy, based on molecular typing of breast cancer, is the most precise form of treatment, and CXC chemokine receptor 2 (CXCR2) is one of the molecular markers used in targeted therapies. As a member of the seven transmembrane G-protein-coupled receptor family, CXCR2 and its associated ligands have been increasingly implicated in tumor-associated processes. These processes include proliferation, angiogenesis, invasion, metastasis, chemoresistance, and stemness and phenotypic maintenance of cancer stem cells. Thus, the inhibition of CXCR2 or its downstream signaling pathways could significantly attenuate tumor progression. Therefore, studies on the biological functions of CXCR2 and its association with neoplasia may help improve the prognosis of breast cancer. Furthermore, the targeting of CXCR2 could supplement the present clinical approaches of breast cancer treatment strategies. The present review discusses the structures and mechanisms of CXCR2 and its ligands. Additionally, the contribution of CXCR2 to the development of breast cancer and its potential therapeutic benefits are also discussed.

## 1. Introduction

Breast cancer is an aggressive malignancy, and is a major threat to the health of women worldwide. According to the 2018 Global Cancer Statistics, breast cancer is the second leading cause of cancer-associated mortality worldwide, following lung cancer. Furthermore, there were ~2.1 million new cases in 2018 worldwide, and these accounted for approximately a quarter of the total number of female patients with cancer (1). In China, breast cancer is the fifth leading cause of cancer-associated mortality, according to statistics collected by the China National Cancer Center in 2018 (2). On the therapy options for breast cancer, molecular typing is a critical basis. Based on the expression of estrogen receptor (ER), progesterone receptor (PR), human epidermal growth factor receptor 2 (HER2) and Ki-67, there are five primary molecular subtypes of breast cancer: Luminal A, luminal B, triple-negative/basal like, HER2-enriched and normal-like (3). Currently, molecular typing-based integrative treatment regimens consist of surgery, radiotherapy, chemotherapy, endocrine therapy and targeted therapy, which have improved treatment efficacy, including improving overall survival (OS) and progression-free survival (4,5). However, most patients with cancer still experience drug resistance, recurrence and metastasis (6). In recent years, CXC chemokine receptor 2 (CXCR2) has emerged as a critical functional receptor. CXCR2 serves an important role in various aspects of breast cancer, including the diverse range of pathological processes associated with tumor progression (7). Combined analysis demonstrated that patients with solid tumors and elevated CXCR2 expression had poorer prognosis, including OS, recurrence-free survival and disease-free survival (8). The present review summarizes the biological roles of CXCR2 in breast cancer and systematically examines the pathways and mechanisms of CXCR2 associated with the initiation and development of breast cancer, as well as the potential therapeutic value of an anti-CXCR2 treatment.

## 2. Structure and interactions of the chemokine CXC and its receptor CXCR2

Chemokines are small (6–14 kDa), secreted peptides that mediate the migration of leukocytes to inflammation and secondary lymphoid organs. Chemokines are also essential for other pathophysiological processes, including infectious diseases, asthma, and atherosclerosis (9–11). Based on the position of the four cysteine (Cys) residues at the N-terminus, the chemokines are divided into four subtypes: CXC (α), CC (β), XC (γ) and CX3C (δ) (12). To date, 50 chemokines and 20 chemokine receptors have been identified; the majority of chemokines belong to the CC and CXC subgroups (13). CXC is further classified into ELR^+^ and ELR^−^ CXC chemokines, based on the glutamate-leucine-arginine (Glu-Leu-Arg, ELR) sequence that occurs before the first Cys at the N-terminus (14).

The coding sequence of the CXCR2 gene is located at 2q34-35 and contains three exons and two introns (15). CXCR2 is a G protein-coupled receptor that contains seven transmembrane regions, an extracellular N-terminus and an intracellular C-terminus (16). The N-terminus, the fourth transmembrane domain and the second extracellular loop are prerequisites for ligand binding and specificity, and determine the rate of receptor internalization (17). The C-terminus region is involved in receptor phosphorylation, internalization and G protein coupling. Only ligand monomers activate CXCR2, which interact via a two-site, two-step model. This model involves the binding of the N-terminal domain of CXCR2 with the N-loop and core domain of ligands at site 1. At site 2, CXCR2 activation is conferred by the insertion of the N-terminus signal domain of the ligands into the orthosteric pocket of the receptor (18). Interleukin-8 (IL-8), also termed CXCL8, is a CXCR2 ligand and was the first CXC chemokine derived from the medium of lipopolysaccharide and polyhydroxyalkanoate-stimulated human monocytes (19). In humans, CXCR2 is also known as IL-8 receptor B and interacts with ELR^+^CXC chemokines with high affinity. These chemokines include GRO-α/CXCL1, GRO-β/CXCL2, GRO-γ/CXCL3, ENA-78/CXCL5, GCP-2/CXCL6, NAP-2/CXCL7 and IL-8/CXCL8, which mediate angiogenesis (20).

## 3. Signaling pathways of CXCR2 activation

CXCR2 possesses no kinase activity. In addition to being coupled to G proteins, it binds to other proteins, such as G protein coupled receptor kinase 2/6, β-arrestin1/2, adaptor protein-2, protein phosphatase 2A and vasodilator-stimulated phosphoprotein. This enables CXCR2 to mediate different signaling cascades in breast cancer (21). G proteins are heterotrimeric protein complexes that are comprised of three subunits, known as α, β and γ, which are inactive in their resting state. Upon binding of the ligands to CXCR2, CXCR2 physically couples to the G protein (22). CXCR2 becomes subsequently activated and the guanosine disphosphate linked to the Gα subunit of the G protein complex is converted to GTP. This transformation causes Gα to dissociate from the receptor and Gβγ, leading to the activation of several downstream signaling pathways.

**Figure 1.**
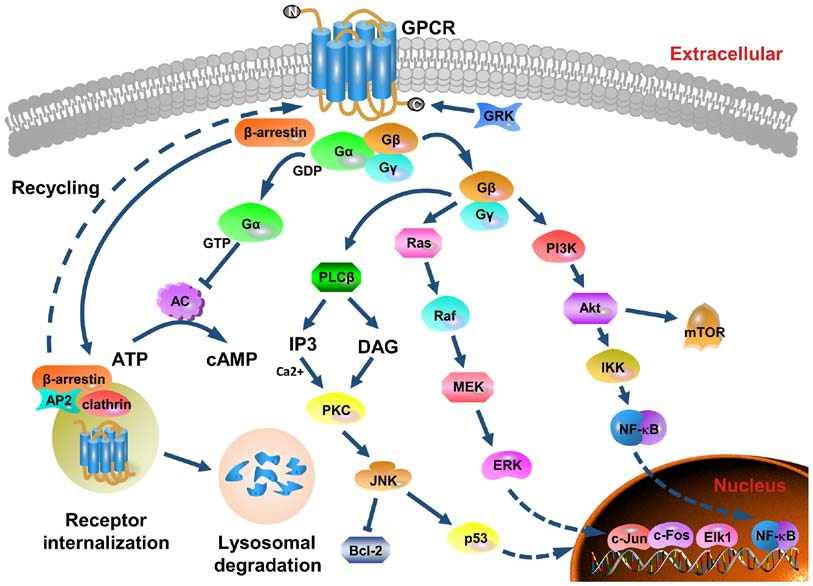
Structure, major signaling cascades and receptor recycling of CXCR2. CXCR2 belongs to the G protein-coupled receptor family that possesses seven transmembrane structures. It contains an extracellular N-terminus, an intracellular C-terminus, three extracellular loops and three cytoplasmic loops. Following ligand binding, CXCR2 physically couples to the G protein. Subsequently, CXCR2 is activated and the GDP linked to the Gα subunit of the G protein complex is converted to GTP. The Gα subunit coupled to the inner cell membrane dissociates from CXCR2 and the Gβγ subunits. Several downstream pathways are induced, and the main three signaling pathways are via PI3K/Akt, PLC/PKC and Ras/Raf/ERK1/2. Moreover, the Gα subunit inhibits adenylate cyclase activity and decreases the efficiency of ATP conversion to cAMP. GRK phosphorylates the C-terminus of the receptor, and mediates the desensitization and endocytosis of the receptor via β-arrestin recruitment of endocytic components. AP-2 also regulates CXCR2 internalization and sequestration. Internalized CXCR2 is subjected to degradation by lysosomes, or recycled to the outer membrane surface. These pathways modulate cell metabolism, survival, proliferation, apoptosis, angiogenesis, transduction and motility. As a positive feedback loop to enhance CXCR2 functionality is formed by upregulating the expression of cytokines and chemokines. CXCR2, CXC chemokine receptor 2; PI3K, phosphatidylinositol-3 kinase; PLC, phospholipase C; PKC, protein kinase C; ERK, extracellular signal related kinase; cAMP, cyclic AMP; GRK, G-protein coupled receptor kinase; GPCR, G-protein coupled receptor; GDP, guanosine disphosphate; AC, adenylyl cyclase.

As presented in Fig. 1, the three main pathways activated by CXCR2 are the phosphatidylinositol-3 kinase (PI3K)/Akt pathway, the phospholipase C (PLC)/protein kinase C (PKC) pathway and the Ras/Raf/extracellular signal related kinases (ERK1/2) pathway (23–25). The PI3K/Akt pathway is the primary downstream signaling cascade mediated by PI3K, and protein kinase B (PKB) plays an essential role in this pathway. PKB is the oncogene product of the retrovirus Aktδ, which is also known as Akt. Akt activates IκB kinase (IKK), which phosphorylates IκB to expose the nuclear localization signals of NF-κB. This allows NF-κB subunits to translocate to the nucleus (26). The PI3K/Akt pathway is one of the most commonly altered pathways in human malignant tumors, and it is critical for cell survival, motility and angiogenesis (27). The phosphorylation of CXCR2 also results in the activation of mitogen-activated protein kinases (MAPK) signaling, which includes ERK. Ras activates ERK through Raf and MEK, and phosphorylated ERK translocates from the cytoplasm to the nucleus. This mediates the transcriptional activation of c-Jun, c-Fos, Elk-1, AP-1 and ATF, which participate in various biological functions, such as cell proliferation and differentiation, morphology maintenance, cytoskeleton construction, apoptosis and tumorigenesis (28,29). The activation of PLC induces another signaling transduction pathway that generates two secondary messengers, inositol triphosphate and diacylglycerol. This leads to calcium mobilization from the endoplasmic reticulum and activates PKC. Subsequently, c-Jun N-terminal kinase influences cell apoptosis by mediating the activity of p53 and Bcl-2 (30,31). Additionally, the release of the Gα subunit from the G protein trimer inhibits the conversion of ATP into cyclic AMP by adenylate cyclase, and this decreases the intracellular levels of cyclic AMP (21). Furthermore, CXCR2 signaling could trigger the pathways of focal adhesion kinase (FAK), Rho, Rac, the Janus kinase/signal transducer and the activator of the transcription pathways (32–34).

These pathways act as modulators in breast cancer cell metabolism, survival, proliferation, apoptosis, angiogenesis, transduction and motility. These form a positive feedback loop to enhance CXCR2 functionality by upregulating the expression of cytokines and chemokines, such as CXCL8 (19).

## 4. Biological characteristics of CXCR2 in breast cancer

### 

#### CXCR2 and breast cancer growth

Tumorigenesis is a process that involves multiple genes, several steps and various signaling pathways, including the complex regulatory network of chemokines and their receptors (35,36). Studies have found that the CXCL8-CXCR2 axis is closely associated with multiple stages of breast cancer growth. These include the regulation of transcription of CDK inhibitors p21Cip1, p27Kip1 and p57Kip2, and the induction of tumor cell proliferation, differentiation, stress response and apoptosis (15,20,37). Paradoxically, CXCL8 enhances the immune system response and increases its ability to execute anti-tumor effects, whereas it also transforms the tumor microenvironment to promote tumor growth. Compared with healthy volunteers, patients with breast cancer have elevated levels of CXCL8, and the severity of this overexpression is positively correlated with disease stage (38). The levels of CXCR2 in malignant tissues are higher than those in benign and normal tissues (39). Polymorphisms in the CXCL8 and CXCR2 genes also suggest that elevated CXCL8 and CXCR2 expression may be risk factors of breast cancer (38).

However, the biological significance of CXCR2 in cancer cell proliferation remains controversial. Shao *et al* (40) performed small interfering RNA-mediated knockdown of endogenous CXCL8 that upregulated p27Kip21 and downregulated cyclin D1. The decreased Akt phosphorylation and NF-κB activation resulted in reduced cell proliferation in both MDA-MB-231 and BT549 breast cancer cell lines. This indicated that CXCL8 and CCL2 overexpression enhances tumor proliferation (40). By contrast, other studies have shown that the overexpression of CXCR2 induces premature senescence, and silencing of CXCR2 prolongs cell passage via p53, NF-κB or C/EBPβ-associated pathways (39,41). Overall, several studies have reported that CXCR2 is a tumor-stimulating receptor that could be exploited as a marker of poor prognosis in a variety of cancer types. Thus, inhibiting CXCR2 production may promote cancer cell apoptosis (42,43). Therefore, CXCR2 may have different functions in normal, precancerous and tumor cells and requires further investigation.

In the tumor microenvironment, breast cancer growth in both autocrine and paracrine manners are regulated by CXCR2 and its ligands produced by stromal cells (44). Furthermore, neutrophils, myeloid cells and bone marrow-derived suppressor cells express CXCR2 and assist in tumor cell proliferation (44). Following the entry of neutrophils into the tumor site, an increase in cytokine secretion contributes to the production of an inflammatory microenvironment (45). Additionally, bone marrow-derived suppressor cells differentiate into M2-type macrophages, which facilitate cancer cell growth (46). Previous studies have demonstrated the knockout of the CXCR2 gene in host cells to inhibit tumor growth and increased tumor cell apoptosis (47–49).

#### CXCR2 and breast cancer angiogenesis

Once tumors exceed 1–2 mm in diameter, angiogenesis is initiated for growth and metastasis (50,51). CXCR2 affects angiogenesis in breast cancer primarily by interacting with CXCL8 and CXCL1, however the specific mechanism is yet to be determined (52–54).

Addison *et al* (53) detected the expression of CXCR2 using a CXCR2 antibody in human microvascular endothelial cells and confirmed that the chemotaxis of ELR^+^CXC chemokine-mediated microvascular endothelial cell was obstructed, and was sensitive to pertussis toxins (53). Studies in CXCR2-deficient mice indicated that CXCL8 is the strongest ligand for CXCR2, and is mediated by the activation of the ELR^+^CXC chemokine (52). In cancer cells, CXCL8 and vascular endothelial growth factor (VEGF) cooperate to establish and expand tumor neovascularization. Furthermore, glucose deprivation and endoplasmic reticulum stress effectively induce the upregulation of CXCL8 (55). CXCL8 and VEGF are regulated by distinct pathways in different cell lines. MDA-MB-231 cells mainly activates the MAPK-ERK pathway, and the activity of the PI3K/Akt pathway is increased in GI101A cells. Both signaling pathways are activated in MDA-MB-468 and Hs578T cell lines (56). CXCL8 generated by endothelial cells binds to CXCR2 to mediate interactions between CXCR2 and VEGFR receptor 2 (VEGFR2). This includes the transactivation of VEGFR2 via Src kinase-mediated receptor phosphorylation, which is required for CXCL8 to induce endothelial cell permeability (56). The CXCL8-CXCR2 axis also induces VEGF transcription and stimulates VEGFR2 activation through the NF-κB pathway in endothelial cells (57). Moreover, the CXCL8-CXCR2 axis activates the expression of EGFR to mediate endothelial cell migration and capillary formation (58). It also elevates integrin αvβ3 levels, which serve a key role in endothelial cell survival and cancer cell migration during tumor angiogenesis (59). Another study revealed that the expression of CXCL8 in ER^+^ cells was lower than that in ER^−^ cells, and exogenous ERα substantially interfered with CXCL8 expression. This suggests that the inactivation of ERα and upregulation of CXCL8 could promote angiogenesis in human breast cancer (60). The silencing of CXCR2 further indicated the importance of CXCL8-mediated angiogenesis. Nannuru *et al* (61) analyzed the microvessel density of primary tumor sections, and found that silencing CXCR2 in Cl66 cells considerably decreased tumor angiogenesis compared with the control group.

Furthermore, thrombin stimulates tumors to secrete CXCL1 in endothelial cells, which reinforces tumor angiogenesis. Thus, thrombin-induced angiogenesis could be perturbed by the CXCL1 antibody (54). In 4T1 cells, shRNA-knockdown of CXCL1 impeded tumor growth and angiogenesis (54).

#### CXCR2 and breast cancer metastasis

Metastasis is a basic biological characteristic of malignant neoplasia. Distant metastasis confers breast cancer a worse prognosis, with the five-year survival rate of 27% in the United States between 2008 and 2014, whereas the five-year survival rate of the localized stage was of 99% (62). Metastasis occurs predominantly through the lymphatic system, blood, direct infiltration and planting. This process is extremely complex, dynamic and continuous, and contains several independent processes. For example, when tumors metastasize via the blood circulation, the cancer cells proliferate within the primary lesion and form new blood vessels. Subsequently, the aggressive cells detach from the primary tumor. This is followed by epithelial-mesenchymal transition (EMT), which results in cells with properties similar to interstitial cells. Epithelial cells subjected to EMT, lose their intercellular connections and polarity, and experience changes in cell morphology and increased migration capacity. Tumor cells grow into the surrounding interstitial space by infiltration, and are in close contact with local capillaries and lymphatic endothelial cells. Once cells have invaded this interstitial space, they subsequently penetrate the walls of the vessel and protrude into the lumen. This enables transport to the target tissue, where the cells then proliferate in the matrix to form a new secondary tumor (63,64).

CXCR2 is involved in the migration, invasion and metastasis of breast cancer cells in various ways (61). A number of studies have investigated the effects of CXCL8 on cancer invasion and migration (65–68). High levels of CXCL8 expression promote angiogenesis and attract neutrophils to release enzymes involved in tissue remodeling and tumor formation. Thus, ectopic expression of CXCL8, stimulated by IL-1β and TNF-α, could exacerbate the metastatic potential of breast cancer (65). A CpG island located upstream of the CXCL8 promoter in the highly metastatic cell lines MDA-231 and MDA-345, results in the upregulation of CXCL8 expression (66). However, the loss of CXCL8 production in MDA-MB-231 and BT549 cells consequently attenuates migration and invasion, which may be due to decreased integrin β3 transcription (40). COX-2-mediated CXCL8 production in ER-negative breast cancer cells promotes osteoclast formation and bone metastasis (67). Compared with patients with no metastasis, those with breast cancer and bone metastases demonstrate increased CXCL8 levels. Furthermore, there is a significant positive correlation between plasma CXCL8 levels and bone resorption (68). In addition, CXCL8 regulates the actin cytoskeleton through Ca^2+^-activated PLC-dependent PKC and Rho-GTPase (69). Additionally, CXCL8 also promotes cell migration through the stimulation of the intracellular Akt pathway (27). Triple-negative breast cancer (TNBC) cells secrete CXCL8, which activates CXCR2 in tumor-associated fibroblasts and tumor-associated macrophages. This results in STAT3 phosphorylation and upregulation of CXCL8 transcription and translation. The binding of CXCL8 to CXCR2 in cancer cells facilitates metastasis (70), which was also observed in MDA-MB-231 cells and xenograft mouse models with CXCR2-knockout, as well as decreased migration, compared with wild-type cells (70). These data indicate that the CXCR2-CXCL8 axis is multifaceted in tumor progression and metastasis, and renders cancer cells invasive.

Bone marrow-derived mesenchymal stem cells (MSCs) express CXCL1 and CXCL5 to recruit PyMT breast cancer cells and prompt the migration in a CXCR2-dependent manner *in vitro*. The CXCR2-inhibitory antibody, SB265610, substantially curbs the migration of cancer cells to MSC-conditioned media (71). Additionally, it was proposed that TNFα-activated MSCs secrete CXCR2 ligands to recruit CXCR2^+^ neutrophils to tumor sites (72). Tumor-associated neutrophils stimulate metastasis through matrix metalloproteinases and other soluble molecules. A co-culture system, consisting of tumor cells and neutrophils, substantiated to higher expression levels of metastasis-associated genes in tumor cells, which identified an MSCs/neutrophil/tumor cell axis associated with cancer metastasis (72).

The silencing of CXCR2 in breast cancer cell lines using short hairpin RNA results in attenuated cell invasion. Moreover, when these shRNA-treated cells were transplanted into an orthotopic mouse xenograft model, spontaneous lung metastases were decreased by 40% compared with the control group (61). In addition, the loss of CXCR2 expression in stroma cells (neutrophils, macrophages and endothelial cells) in the tumor microenvironment also prevented cancer cell migration (47). It was hypothesized that CXCR2 is involved in the paracrine loop between tumor cells and its surroundings, which potentiates invasion and metastasis.

#### CXCR2 and drug resistance in breast cancer

Drug resistance is one of the challenges of advanced breast cancer therapy. Tumors gradually become less responsive to chemoradiotherapy during treatment and therefore, the mortality rate of breast cancer remains high. As a consequence of drug resistance, there are few recognized therapeutic strategies (73,74).

A wealth of evidence has shown that malignant cells that survive primary chemoradiotherapy express higher levels of CXCR2 ligands (65,75). Additionally, high levels of CXCL1, CXCL3, CXCL5, CXCL6, CXCL7 and CXCL8 are observed in drug-resistant breast cancer cells, which diminishes the effectiveness of other medical interventions (7,76). Shi *et al* (76) revealed that the multidrug-resistant human breast cancer cell line, MCF-7/R, has higher expression levels of CXCL6 and CXCL8 compared with the sensitive control cell line MCF-7/S. The inhibition of CXCL6 and CXCL8 with antibodies enhances the sensitivity to paclitaxel and doxorubicin treatment in MCF-7/R cells. The inhibition of CXCL6 and CXCL8 expression reverses the chemoresistance of MCF-7/R cells, and the overexpression of CXCL6 and CXCL8 potentiates the resistance of MCF-7/S cells to doxorubicin (76). Similarly, other studies demonstrated that the inhibition of CXCL8 in MDA-MB-231 and BT549 cells improved the efficacy of chemotherapy (40). The inhibition of IL-8 or CXCR2 was shown to prevent the paclitaxel-induced autocrine inflammatory feed-forward loop (77). Sharma *et al* (75) used Cl66-wt, 4T1-wt, Cl66sh-CXCR2 and 4T1sh-CXCR2 cells that expressed varying levels of CXCR2, to assess the effect of CXCR2 levels on chemosensitivity. Furthermore, it was reported that the silencing of CXCR2 could increase the cytotoxicity of paclitaxel and doxorubicin, and intensify the antitumor activity of these drugs (75).

Regarding the intracellular mechanism of CXCR2-mediated drug resistance, previous studies suggested that AKT regulates chemical resistance through a broad anti-apoptotic molecule, such as PED (78–80). Patients with breast cancer and Akt phosphorylation at serine 473 are more sensitive to paclitaxel treatment (81). The overexpression of COX2 results in chemical resistance to breast cancer by generating prostaglandin H2 and activating NF-κB (82), which was also observed in a study by Xu *et al* (39). These results suggest that CXCR2-dependent regulatory pathways are crucial for cancer cells to maintain chemoresistance, and provide evidence that supports the targeting of the CXCR2 axis as an adjunctive therapy to prevent drug resistance.

#### CXCR2 and breast cancer stem cells

Cancer stem cells (CSCs) were first identified in breast cancer (83), and the presence of CSCs may be responsible for the high level of heterogeneity observed in this disease (84). Breast cancer stem cells (BCSCs) are resistant to the effects of chemoradiotherapy and endocrine therapy due to their self-renewal and differentiation potential. This allows BCSBCs to initiate and maintain tumor growth, invasion, metastasis, drug resistance and recurrence (85). The importance of CSCs was demonstrated in several cancer types (86). Trastuzumab prolongs survival in patients with HER2-positive breast cancer treatment, which is in part due to its ability to limit CSCs (87). Neoadjuvant lapatinib was shown to suppress BCSCs in HER2-positive tumors and its combination with lapatinib prolongs the time to progression in patients with trastuzumab resistance (88).

CXCL8 transmits signals through CXCR2, which induces EMT (89). EMT is a process that regulates invasion and metastasis, and enables cells to obtain stem cell characteristics (36). Singh *et al* (90) demonstrated a positive correlation between the level of CXCL8 in metastatic pleural and peritoneal effusion and the ability of these tumor cells to form mammospheres *in vitro*. The inhibition of CXCR2 using a small molecule CXCR1/2 antagonist, SCH563705, abolished the impact of CXCL8 expression. This was quantified by enzyme-linked immunosorbent assay (90). The suppression of CXCR2 expression limits tumor spheroid formation and aldefluor-positive rate in breast cancer cells, and increases the efficacy of anti-HER2 therapy in HER2-positive patients (91). Furthermore, CXCR2 regulates the activity of CSCs through both HER2-dependent and HER2-independent pathways. One such pathway is through FAK, which is also associated with BCSC maintenance (92). *In vivo* studies have shown that MSCs are recruited to the tumor site, co-localized with CSCs in the microenvironment and secreted cytokines. This increases the number of CSCs present in the tumor, thereby accelerating tumor growth (93). Liu *et al* (93) suggested that this effect is initiated by CXCL6 secreted by cancer cells, and is maintained by CXCL8 and other CXCR2 ligands (GCP-2 and NAP-2) that are secreted by cancer cells and stromal cells. In addition, cancer cell-derived CXCL1 induces the expression of CXCL8 and GRO-α in MSCs, which promotes the formation and maintenance of CSCs (94). The inhibition of CXCR2 or downstream pathways can decrease the number and activity of CSCs *in vitro* and in xenografts, and increase the efficacy of docetaxel to decrease tumor volume (95). Recently, studies have identified CXCR2 as a novel BCSC biomarker of only TNBC. CXCR2 was observed to be co-expressed with CSC-associated proteins, such as NANOG and SOX2 (96,97). Furthermore, 4T1 cells that express CXCR2 have characteristics of CSCs, including a low proportion of CXCR2-positive cells (~1%), hypoxia, elevated expression of CSC-associated mRNA, increased tumor spherule formation, tumor xenografts, resistance to radiotherapy and chemotherapy (96).

In addition to CXCR2, CXCR1 was also found to be present on the surface of BCSCs, which facilitates the growth of BCSCs stimulated by tissue damage or inflammation (98). CXCR1 is also known as IL-8 receptor A, and is an active receptor selectively expressed by BCSCs (99). CXCR1 belongs to the same chemokine CXC receptor family as CXCR2, and CXCR1 has 75% sequence similarity with CXCR2 (100). It was proposed that CXCR1 has a prominent role in both the initiation and therapy of breast cancer (101). The inhibition of CXCR1 results in the depletion of BCSCs *in vitro* (102). CXCR1 inhibition combined with chemotherapy, results in the release of CXCL8 by dying bulk (non-CSC) tumor cells and binds with CXCR1 on the surface of BCSCs. As a result, the BCSCs are protected from apoptotic signals triggered by the Fas ligand (102). Several CXCR2 inhibitors, such as Reparixin, also block CXCR1. This further decreases the enrichment of BCSCs and prevents tumor recurrence (77,79).

The aforementioned results uncover the integral role played by CXCR2 signaling in the complex inflammatory cytokine response. Furthermore, the role of CXCR1 in maintaining the activity of BCSCs via autocrine or paracrine pathways, and its effects on treatment efficacy and disease prognosis have been uncovered (87).

## 5. Preclinical and clinical evidence of targeting CXCR2 in breast cancer

**Figure 2.**
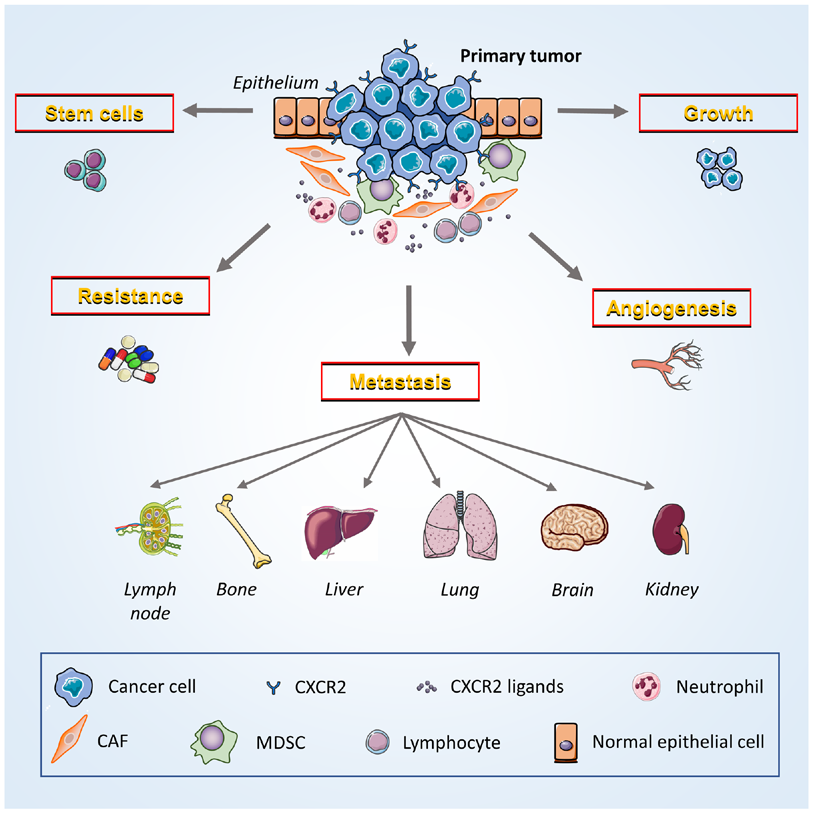
Potential contribution of CXCR2 to breast cancer development and progression. Breast cancer cells and multiple host cells (MDSCs, neutrophils and lymphocytes) in the tumor microenvironment exert various cancer-promoting functions that are induced by the interaction of CXCR2 and its ligands. Tumor cells express both CXCR2 and specific ligands to promote the growth of neoplasms via autocrine and paracrine signaling. CXCR2 also facilitate crosstalk with vascular endothelial cells, induce angiogenesis and enhance the ability of cancer cells to invade and migrate. CXCR2 promotes the metastasis of cancer cells to other locations throughout the body, including lymph nodes, bones, liver, lungs, brain and kidneys. Moreover, CXCR2 decreases the sensitivity of cancer cells to chemoradiotherapy by enabling them to survive treatment. Furthermore, the maintenance of cancer stem cell activity may depend on the ability of CXCR2 to induce the stemness of breast cancer cells. CXCR2, CXC chemokine receptor 2; MDSCs, myeloid-derived suppressor cells; CAFs, cancer-associated fibroblasts.

Targeted therapy offers a unique opportunity to suppress the activity of key genes associated with tumorigenesis (103). CXCR2 and its ligands are influential targets involved in tumor growth and regulation (Fig. 2) (19). Additionally, preclinical studies on other malignant neoplasms have revealed that targeting CXCR2 alone, or in combination with other regimens, may provide an effective, novel option for cancer management (104–107).

Although CXCL8 is the most commonly studied ligand of CXCR2 in breast cancer, other CXCR2 receptor agonists, such as GRO-α, GRO-β, GRO-γ and CXCL5, are co-regulated with CXCL8. This co-regulation of CXCR2 ligands may limit the effectiveness of targeting CXCL8 alone (108), which could be circumvented by suppressing CXCR2 and its downstream signaling pathways. Blocking the function of CXCR2, by impeding ligand binding or subsequent pathway activation, essentially prevents the biological effects of multiple ELR^+^ CXC chemokines with a single treatment. This intervention may be more tumor-specific than the direct inhibition of cytokines, and could decrease the severity of adverse events.

There are various orally active small-molecules that are non-competitive antagonists of CXCR2, including Repertaxin, SCH479833, SCH527123 and SCH563705. These antagonists exhibit anti-tumor properties in breast cancer (95), colorectal cancer (105) and melanoma (106) xenograft models. These properties include the obstruction of spontaneous hepatic metastasis of colon carcinomas (109). Moreover, the combination of paclitaxel treatment and the inhibition of CXCR2 slows breast tumor growth, attenuates tumor angiogenesis and lung metastasis, and diminishes the number of CSCs. Additionally, targeting CXCR2 reduces drug resistance (75,110). Since CXCL8 is a prominent regulator of BCSC activity, a combined treatment of CXCR2 inhibitors and current HER2-targeted therapies is predicted to be an effective regimen to decrease CSC activity and increase survival in HER2-positive patients with breast cancer (90). A recent study in pancreatic ductal adenocarcinomas demonstrated that inhibition of CXCR2 predominantly in neutrophils/MDSCs confers T cell entry to the tumor site (111). The inhibition of CXCR2 also enhances the sensitivity to anti-programmed death 1 therapy, which reinforces the endogenous anti-tumor immune response (111). Moreover, Uddin *et al* (98) revealed that the inhibition of proteasome significantly upregulates the levels of CXCL8 and its receptors CXCR1/2, through IKK. The inhibition of the induced CXCL8 expression or IKK activity may improve the effectiveness of proteasome inhibitors as a therapy for TNBC. Overall, CXCR2 and its ligands are potential therapeutic targets.

Due to the various functions of CXCL8 in homeostatic processes, the side effects of CXCR2 inhibitors cannot be ignored. Neutrophils are part of the immune surveillance system that monitors and destroys transformed cells, and CXCL8 exerts an anti-tumor effect by recruiting concentrated granulocytes (112). Therefore, the anti-tumor effects of CXCR2 inhibitors that influence neutrophil infiltration may unintentionally promote tumor growth. The solution to this dilemma depends on the new technologies of targeted drug therapies for cancer cells. In addition, CXCR2 inhibitors cause a marked decrease in the number of circulating neutrophils combined with chemotherapeutic agents, and this could cause a potentially synergistic increase in myelotoxicity (113).

Currently, CXCR2 antagonists are used in phase II clinical trials for chronic obstructive pulmonary disease (114), and obvious adverse reactions were reported in patients with severe asthma (113). Repertaxin was originally used to prevent CXCL8-induced reperfusion injury, and was the only clinically-tested CXCR2 inhibitor (21). Interfering with CXCR2 is particularly advantageous in breast cancer compared with other tumors. Phase I clinical trials have shown that Repertaxin, in combination with paclitaxel, is safe and well tolerated (99). Phase II studies on this combination are currently ongoing (99). The principle data on the efficacy and safety lay an essential foundation for the accelerated development of CXCR2 inhibitors as a treatment for breast cancer.

## 6. Conclusions and outlooks

Currently, controversies still exist in the field of CXCR2 research. One controversy is the association between CXCR2 and the expression of ER. Other controversies include the characterization of CXCR2 in cancer cell senescence, and its role in Akt-associated tumor metastasis (39,115–117). These controversies may be attributed to the limitations of the pathological tissue, cell type and animal model used.

The association between inflammation and tumor development is widely recognized, and the expression of many inflammatory cytokines in the tumor microenvironment has previously been reported (19). The poor efficacy of current breast cancer treatments may be associated with the molecular typing, and the function of CXCR2 in breast cancer. The anti-tumor effect of targeting CXCR2 was reflected in both *in vitro* and *in vivo* models of breast cancer, and further supports the development of CXCR2 inhibitors as a clinical therapy in breast cancer. Further exploration of whether CXCR2 inhibition plays a vital role in targeted therapies for breast cancer, based on molecular typing, is warranted.

## Data Availability

Not applicable.

## References

[1] Bray F, Ferlay J, Soerjomataram I, Siegel RL, Torre LA, Jemal A (2018). Global cancer statistics 2018: GLOBOCAN estimates of incidence and mortality worldwide for 36 cancers in 185 countries. CA Cancer J Clin.

[2] Chen W, Sun K, Zheng R, Zeng H, Zhang S, Xia C, Yang Z, Li H, Zou X, He J (2018). Cancer incidence and mortality in China, 2014. Chin J Cancer Res.

[3] Harbeck N, Thomssen C, Gnant M (2013). St. Gallen 2013: Brief preliminary summary of the consensus discussion. Breast Care (Basel).

[4] Edenfield J, Schammel C, Collins J, Schammel D, Edenfield WJ (2017). Metaplastic breast cancer: Molecular typing and identification of potential targeted therapies at a single institution. Clin Breast Cancer.

[5] Ma F, Guan Y, Yi Z, Chang L, Li Q, Chen S, Zhu W, Guan X, Li C, Qian H (2019). Assessing tumor heterogeneity using ctDNA to predict and monitor therapeutic response in metastatic breast cancer. Int J Cancer.

[6] Lee KL, Kuo YC, Ho YS, Huang YH (2019). Triple-negative breast cancer: Current understanding and future therapeutic breakthrough targeting cancer stemness. Cancers.

[7] Sharma B, Varney ML, Saxena S, Wu L, Singh RK (2016). Induction of CXCR2 ligands, stem cell-like phenotype and metastasis in chemotherapy-resistant breast cancer cells. Cancer Lett.

[8] Yang Y, Luo B, An Y, Sun H, Cai H, Sun D (2017). Systematic review and meta-analysis of the prognostic value of CXCR2 in solid tumor patients. Oncotarget.

[9] Murdoch C, Finn A (2000). Chemokine receptors and their role in inflammation and infectious diseases. Blood.

[10] Liang Y, Feng Y, Wu W, Chang C, Chen D, Chen S, Zhen G (2019). MicroRNA-218-5p plays a protective role in eosinophilic airway inflammation via targeting δ-catenin, a novel catenin in asthma. Clin Exp Allergy.

[11] Wang X, Iyer A, Lyons AB, Körner H, Wei W (2019). Emerging roles for G-protein coupled receptors in development and activation of macrophages. Front Immunol.

[12] Rollins BJ (1997). Chemokines. Blood.

[13] Debnath B, Xu S, Grande F, Garofalo A, Neamati N (2013). Small molecule inhibitors of CXCR4. Theranostics.

[14] O'Hayer KM, Brady DC, Counter CM (2009). ELR+ CXC chemokines and oncogenic Ras-mediated tumorigenesis. Carcinogenesis.

[15] Lloyd A, Modi W, Sprenger H, Cevario S, Oppenheim J, Kelvin D (1993). Assignment of genes for interleukin-8 receptors (IL8R) A and B to human chromosome band 2q35. Cytogenet Cell Genet.

[16] Kobilka BK (2007). G protein coupled receptor structure and activation. Biochim Biophys Acta.

[17] Prado GN, Suetomi K, Shumate D, Maxwell C, Ravindran A, Rajarathnam K, Navarro J (2007). Chemokine signaling specificity: Essential role for the N-terminal domain of chemokine receptors. Biochemistry.

[18] Moussouras NA, Getschman AE, Lackner ER, Veldkamp CT, Dwinell MB, Volkman BF (2017). Differences in sulfotyrosine binding amongst CXCR1 and CXCR2 chemokine ligands. Int J Mol Sci.

[19] Cheng Y, Ma XL, Wei YQ, Wei XW (2019). Potential roles and targeted therapy of the CXCLs/CXCR2 axis in cancer and inflammatory diseases. Biochim Biophys Acta Rev Cancer.

[20] Ahuja SK, Murphy PM (1996). The CXC chemokines growth-regulated oncogene (GRO) alpha, GRObeta, GROgamma, neutrophil-activating peptide-2, and epithelial cell-derived neutrophil-activating peptide-78 are potent agonists for the type B, but not the type A, human interleukin-8 receptor. J Biol Chem.

[21] Ha H, Debnath B, Neamati N (2017). Role of the CXCL8-CXCR1/2 axis in cancer and inflammatory diseases. Theranostics.

[22] Damaj BB, McColl SR, Mahana W, Crouch MF, Naccache PH (1996). Physical association of Gi2alpha with interleukin-8 receptors. J Biol Chem.

[23] Wu D, LaRosa GJ, Simon MI (1993). G protein-coupled signal transduction pathways for interleukin-8. Science.

[24] Knall C, Young S, Nick JA, Buhl AM, Worthen GS, Johnson GL (1996). Interleukin-8 regulation of the Ras/Raf/mitogen-activated protein kinase pathway in human neutrophils. J Biol Chem.

[25] Knall C, Worthen GS, Johnson GL (1997). Interleukin 8-stimulated phosphatidylinositol-3-kinase activity regulates the migration of human neutrophils independent of extracellular signal-regulated kinase and p38 mitogen-activated protein kinases. Proc Natl Acad Sci USA.

[26] Oeckinghaus A, Ghosh S (2009). The NF-kappaB family of transcription factors and its regulation. Cold Spring Harb Perspect Biol.

[27] Cheng GZ, Park S, Shu S, He L, Kong W, Zhang W, Yuan Z, Wang LH, Cheng JQ (2008). Advances of AKT pathway in human oncogenesis and as a target for anti-cancer drug discovery. Curr Cancer Drug Targets.

[28] Hoffmann E, Dittrich-Breiholz O, Holtmann H, Kracht M (2002). Multiple control of interleukin-8 gene expression. J Leukoc Biol.

[29] Tang H, Sun Y, Shi Z, Huang H, Fang Z, Chen J, Xiu Q, Li B (2013). YKL-40 induces IL-8 expression from bronchial epithelium via MAPK (JNK and ERK) and NF-κB pathways, causing bronchial smooth muscle proliferation and migration. J Immunol.

[30] Clapham DE (1995). Calcium signaling. Cell.

[31] Lang K, Niggemann B, Zanker KS, Entschladen F (2002). Signal processing in migrating T24 human bladder carcinoma cells: Role of the autocrine interleukin-8 loop. Int J Cancer.

[32] Schraufstatter IU, Chung J, Burger M (2001). IL-8 activates endothelial cell CXCR1 and CXCR2 through Rho and Rac signaling pathways. Am J Physiol Lung Cell Mol Physiol.

[33] Cohen-Hillel E, Yron I, Meshel T, Soria G, Attal H, Ben-Baruch A (2006). CXCL8-induced FAK phosphorylation via CXCR1 and CXCR2: Cytoskeleton- and integrin-related mechanisms converge with FAK regulatory pathways in a receptor-specific manner. Cytokine.

[34] Britschgi A, Andraos R, Brinkhaus H, Klebba I, Romanet V, Müller U, Murakami M, Radimerski T, Bentires-Alj M (2012). JAK2/STAT5 inhibition circumvents resistance to PI3K/mTOR blockade: A rationale for cotargeting these pathways in metastatic breast cancer. Cancer Cell.

[35] Sotiriou C, Neo SY, McShane LM, Korn EL, Long PM, Jazaeri A, Martiat P, Fox SB, Harris AL, Liu ET (2003). Breast cancer classification and prognosis based on gene expression profiles from a population-based study. Proc Natl Acad Sci USA.

[36] Hanahan D, Weinberg RA (2011). Hallmarks of cancer: The next generation. Cell.

[37] Hebert CA, Vitangcol RV, Baker JB (1991). Scanning mutagenesis of interleukin-8 identifies a cluster of residues required for receptor binding. J Biol Chem.

[38] Snoussi K, Mahfoudh W, Bouaouina N, Fekih M, Khairi H, Helal AN, Chouchane L (2010). Combined effects of IL-8 and CXCR2 gene polymorphisms on breast cancer susceptibility and aggressiveness. BMC Cancer.

[39] Xu H, Lin F, Wang Z, Yang L, Meng J, Ou Z, Shao Z, Di G, Yang G (2018). CXCR2 promotes breast cancer metastasis and chemoresistance via suppression of AKT1 and activation of COX2. Cancer Lett.

[40] Shao N, Chen LH, Ye RY, Lin Y, Wang SM (2013). The depletion of interleukin-8 causes cell cycle arrest and increases the efficacy of docetaxel in breast cancer cells. Biochem Biophys Res Commun.

[41] Ruan JW, Liao YC, Lua I, Li MH, Hsu CY, Chen JH (2012). Human pituitary tumor-transforming gene 1 overexpression reinforces oncogene-induced senescence through CXCR2/p21 signaling in breast cancer cells. Breast Cancer Res.

[42] Lee YS, Choi I, Ning Y, Kim NY, Khatchadourian V, Yang D, Chung HK, Choi D, LaBonte MJ, Ladner RD (2012). Interleukin-8 and its receptor CXCR2 in the tumour microenvironment promote colon cancer growth, progression and metastasis. Br J Cancer.

[43] Desurmont T, Skrypek N, Duhamel A, Jonckheere N, Millet G, Leteurtre E, Gosset P, Duchene B, Ramdane N, Hebbar M (2015). Overexpression of chemokine receptor CXCR2 and ligand CXCL7 in liver metastases from colon cancer is correlated to shorter disease-free and overall survival. Cancer Sci.

[44] Katoh H, Wang D, Daikoku T, Sun H, Dey SK, Dubois RN (2013). CXCR2-expressing myeloid-derived suppressor cells are essential to promote colitis-associated tumorigenesis. Cancer Cell.

[45] Zhou SL, Dai Z, Zhou ZJ, Wang XY, Yang GH, Wang Z, Huang XW, Fan J, Zhou J (2012). Overexpression of CXCL5 mediates neutrophil infiltration and indicates poor prognosis for hepatocellular carcinoma. Hepatology.

[46] Pollard JW (2004). Tumour-educated macrophages promote tumour progression and metastasis. Nat Rev Cancer.

[47] Sharma B, Nannuru KC, Varney ML, Singh RK (2015). Host Cxcr2-dependent regulation of mammary tumor growth and metastasis. Clin Exp Metastasis.

[48] Singh S, Varney M, Singh RK (2009). Host CXCR2-dependent regulation of melanoma growth, angiogenesis and experimental lung metastasis. Cancer Res.

[49] Cardona AE, Sasse ME, Liu L, Cardona SM, Mizutani M, Savarin C, Hu T, Ransohoff RM (2008). Scavenging roles of chemokine receptors: Chemokine receptor deficiency is associated with increased levels of ligand in circulation and tissues. Blood.

[50] Folkman J (2006). Angiogenesis. Annu Rev Med.

[51] Folkman J (1971). Tumor angiogenesis: Therapeutic implications. N Engl J Med.

[52] Stadtmann A, Zarbock A (2012). CXCR2: From bench to bedside. Front Immunol.

[53] Addison CL, Daniel TO, Burdick MD, Liu H, Ehlert JE, Xue YY, Buechi L, Walz A, Richmond A, Strieter RM (2000). The CXC chemokine receptor 2, CXCR2, is the putative receptor for ELR+ CXC chemokine-induced angiogenic activity. J Immunol.

[54] Caunt M, Hu L, Tang T, Brooks PC, Ibrahim S, Karpatkin S (2006). Growth-regulated oncogene is pivotal in thrombin-induced angiogenesis. Cancer Res.

[55] Marjon PL, Bobrovnikova-Marjon EV, Abcouwer SF (2004). Expression of the pro-angiogenic factors vascular endothelial growth factor and interleukin-8/CXCL8 by human breast carcinomas is responsive to nutrient deprivation and endoplasmic reticulum stress. Mol Cancer.

[56] Petreaca ML, Yao M, Liu Y, Defea K, Martins-Green M (2007). Transactivation of vascular endothelial growth factor receptor-2 by interleukin-8 (IL-8/CXCL8) is required for IL-8/CXCL8-induced endothelial permeability. Mol Biol Cell.

[57] Martin D, Galisteo R, Gutkind JS (2009). CXCL8/IL8 stimulates vascular endothelial growth factor (VEGF) expression and the autocrine activation of VEGFR2 in endothelial cells by activating NFkappaB through the CBM (Carma3/Bcl10/Malt1) complex. J Biol Chem.

[58] Kyriakakis E, Cavallari M, Pfaff D, Fabbro D, Mestan J, Philippova M, De Libero G, Erne P, Resink TJ (2011). IL-8-mediated angiogenic responses of endothelial cells to lipid antigen activation of iNKT cells depend on EGFR transactivation. J Leukoc Biol.

[59] Niu G, Chen X (2011). Why integrin as a primary target for imaging and therapy. Theranostics.

[60] Lin Y, Huang R, Chen L, Li S, Shi Q, Jordan C, Huang RP (2004). Identification of interleukin-8 as estrogen receptor-regulated factor involved in breast cancer invasion and angiogenesis by protein arrays. Int J Cancer.

[61] Nannuru KC, Sharma B, Varney ML, Singh RK (2011). Role of chemokine receptor CXCR2 expression in mammary tumor growth, angiogenesis and metastasis. J Carcinog.

[62] Siegel RL, Miller KD, Jemal A (2019). Cancer statistics, 2019. CA Cancer J Clin.

[63] Welch DR, Hurst DR (2019). Defining the hallmarks of metastasis. Cancer Res.

[64] van der Horst G, Bos L, van der Pluijm G (2012). Epithelial plasticity, cancer stem cells, and the tumor-supportive stroma in bladder carcinoma. Mol Cancer Res.

[65] De Larco JE, Wuertz BR, Rosner KA, Erickson SA, Gamache DE, Manivel JC, Furcht LT (2001). A potential role for interleukin-8 in the metastatic phenotype of breast carcinoma cells. Am J Pathol.

[66] De Larco JE, Wuertz BR, Yee D, Rickert BL, Furcht LT (2003). Atypical methylation of the interleukin-8 gene correlates strongly with the metastatic potential of breast carcinoma cells. Proc Natl Acad Sci USA.

[67] Singh B, Berry JA, Vincent LE, Lucci A (2006). Involvement of IL-8 in COX-2-mediated bone metastases from breast cancer. J Surg Res.

[68] Kamalakar A, Bendre MS, Washam CL, Fowler TW, Carver A, Dilley JD, Bracey JW, Akel NS, Margulies AG, Skinner RA (2014). Circulating interleukin-8 levels explain breast cancer osteolysis in mice and humans. Bone.

[69] Waugh DJ, Wilson C (2008). The interleukin-8 pathway in cancer. Clin cancer Res.

[70] Jin K, Pandey NB, Popel AS (2017). Crosstalk between stromal components and tumor cells of TNBC via secreted factors enhances tumor growth and metastasis. Oncotarget.

[71] Halpern JL, Kilbarger A, Lynch CC (2011). Mesenchymal stem cells promote mammary cancer cell migration in vitro via the CXCR2 receptor. Cancer Lett.

[72] Yu PF, Huang Y, Han YY, Lin LY, Sun WH, Rabson AB, Wang Y, Shi YF (2017). TNFα-activated mesenchymal stromal cells promote breast cancer metastasis by recruiting CXCR2^+^ neutrophils. Oncogene.

[73] Marquette C, Nabell L (2012). Chemotherapy-resistant metastatic breast cancer. Curr Treat Options Oncol.

[74] Chen Y, Zhang Y (2018). Application of the CRISPR/Cas9 system to drug resistance in breast cancer. Adv Sci (Weinh).

[75] Sharma B, Nawandar DM, Nannuru KC, Varney ML, Singh RK (2013). Targeting CXCR2 enhances chemotherapeutic response, inhibits mammary tumor growth, angiogenesis, and lung metastasis. Mol Cancer Ther.

[76] Shi Z, Yang WM, Chen LP, Yang DH, Zhou Q, Zhu J, Chen JJ, Huang RC, Chen ZS, Huang RP (2012). Enhanced chemosensitization in multidrug-resistant human breast cancer cells by inhibition of IL-6 and IL-8 production. Breast Cancer Res Treat.

[77] Jia D, Li L, Andrew S, Allan D, Li X, Lee J, Ji G, Yao Z, Gadde S, Figeys D, Wang L (2017). An autocrine inflammatory forward-feedback loop after chemotherapy withdrawal facilitates the repopulation of drug-resistant breast cancer cells. Cell Death Dis.

[78] Stassi G, Garofalo M, Zerilli M, Ricci-Vitiani L, Zanca C, Todaro M, Aragona F, Limite G, Petrella G, Condorelli G (2005). PED mediates AKT-dependent chemoresistance in human breast cancer cells. Cancer Res.

[79] Festuccia C, Gravina GL, D'Alessandro AM, Millimaggi D, Di Rocco C, Dolo V, Ricevuto E, Vicentini C, Bologna M (2008). Downmodulation of dimethyl transferase activity enhances tumor necrosis factor-related apoptosis-inducing ligand-induced apoptosis in prostate cancer cells. Int J Oncol.

[80] Zanca C, Cozzolino F, Quintavalle C, Di Costanzo S, Ricci-Vitiani L, Santoriello M, Monti M, Pucci P, Condorelli G (2010). PED interacts with Rac1 and regulates cell migration/invasion processes in human non-small cell lung cancer cells. J Cell Physiol.

[81] Yang SX, Costantino JP, Kim C, Mamounas EP, Nguyen D, Jeong JH, Wolmark N, Kidwell K, Paik S, Swain SM (2010). Akt phosphorylation at Ser473 predicts benefit of paclitaxel chemotherapy in node-positive breast cancer. J Clin Oncol.

[82] Zatelli MC, Molé D, Tagliati F, Minoia M, Ambrosio MR, Degli Uberti E (2009). Cyclo-oxygenase 2 modulates chemoresistance in breast cancer cells involving NF-kappaB. Cell Oncol.

[83] Al-Hajj M, Wicha MS, Benito-Hernandez A, Morrison SJ, Clarke MF (2003). Prospective identification of tumorigenic breast cancer cells. Proc Natl Acad Sci USA.

[84] van Nijnatten TJA, Moossdorff M, de Munck L, Goorts B, Vane MLG, Keymeulen KBMI, Beets-Tan RGH, Lobbes MBI, Smidt ML (2017). TNM classification and the need for revision of pN3a breast cancer. Eur J Cancer.

[85] Charafe-Jauffret E, Ginestier C, Iovino F, Wicinski J, Cervera N, Finetti P, Hur MH, Diebel ME, Monville F, Dutcher J (2009). Breast cancer cell lines contain functional cancer stem cells with metastatic capacity and a distinct molecular signature. Cancer Res.

[86] Chen L, Fan J, Chen H, Meng Z, Chen Z, Wang P, Liu L (2014). The IL-8/CXCR1 axis is associated with cancer stem cell-like properties and correlates with clinical prognosis in human pancreatic cancer cases. Sci Rep.

[87] Magnifico A, Albano L, Campaner S, Delia D, Castiglioni F, Gasparini P, Sozzi G, Fontanella E, Menard S, Tagliabue E (2009). Tumor-initiating cells of HER2-positive carcinoma cell lines express the highest oncoprotein levels and are sensitive to trastuzumab. Clin Cancer Res.

[88] Cameron D, Casey M, Press M, Lindquist D, Pienkowski T, Romieu CG, Chan S, Jagiello-Gruszfeld A, Kaufman B, Crown J (2008). A phase III randomized comparison of lapatinib plus capecitabine versus capecitabine alone in women with advanced breast cancer that has progressed on trastuzumab: Updated efficacy and biomarker analyses. Breast Cancer Res Treat.

[89] Fernando RI, Castillo MD, Litzinger M, Hamilton DH, Palena C (2011). IL-8 signaling plays a critical role in the epithelial-mesenchymal transition of human carcinoma cells. Cancer Res.

[90] Singh JK, Farnie G, Bundred NJ, Simões BM, Shergill A, Landberg G, Howell SJ, Clarke RB (2013). Targeting CXCR1/2 significantly reduces breast cancer stem cell activity and increases the efficacy of inhibiting HER2 via HER2-dependent and -independent mechanisms. Clin Cancer Res.

[91] Harrison H, Farnie G, Howell SJ, Rock RE, Stylianou S, Brennan KR, Bundred NJ, Clarke RB (2010). Regulation of breast cancer stem cell activity by signaling through the Notch4 receptor. Cancer Res.

[92] Luo M, Fan H, Nagy T, Wei H, Wang C, Liu S, Wicha MS, Guan JL (2009). Mammary epithelial-specific ablation of the focal adhesion kinase suppresses mammary tumorigenesis by affecting mammary cancer stem/progenitor cells. Cancer Res.

[93] Liu S, Ginestier C, Ou SJ, Clouthier SG, Patel SH, Monville F, Korkaya H, Heath A, Dutcher J, Kleer CG (2011). Breast cancer stem cells are regulated by mesenchymal stem cells through cytokine networks. Cancer Res.

[94] Li HJ, Reinhardt F, Herschman HR, Weinberg RA (2012). Cancer-stimulated mesenchymal stem cells create a carcinoma stem cell niche via prostaglandin E2 signaling. Cancer Discov.

[95] Ginestier C, Liu S, Diebel ME, Korkaya H, Luo M, Brown M, Wicinski J, Cabaud O, Charafe-Jauffret E, Birnbaum D (2010). CXCR1 blockade selectively targets human breast cancer stem cells in vitro and in xenografts. J Clin Invest.

[96] Wang Y, Tu L, Du C, Xie X, Liu Y, Wang J, Li Z, Jiang M, Cao D, Yan X, Luo F (2018). CXCR2 is a novel cancer stem-like cell marker for triple-negative breast cancer. Onco Targets Ther.

[97] Kumar S, Wilkes DW, Samuel N, Blanco MA, Nayak A, Alicea-Torres K, Gluck C, Sinha S, Gabrilovich D, Chakrabarti R (2018). ΔNp63-driven recruitment of myeloid-derived suppressor cells promotes metastasis in triple-negative breast cancer. J Clin Invest.

[98] Uddin MM, Zou Y, Sharma T, Gatla HR, Vancurova I (2018). Proteasome inhibition induces IKK-dependent interleukin-8 expression in triple negative breast cancer cells: Opportunity for combination therapy. PLoS One.

[99] Schott AF, Goldstein LJ, Cristofanilli M, Ruffini PA, McCanna S, Reuben JM, Perez RP, Kato G, Wicha M (2017). Phase Ib pilot study to evaluate reparixin in combination with weekly paclitaxel in patients with HER-2-negative metastatic breast cancer. Clin Cancer Res.

[100] Jones SA, Moser B, Thelen M (1995). A comparison of post-receptor signal transduction events in Jurkat cells transfected with either IL-8R1 or IL-8R2. Chemokine mediated activation of p42/p44 MAP-kinase (ERK-2). FEBS Lett.

[101] Xue MQ, Liu J, Sang JF, Su L, Yao YZ (2017). Expression characteristic of CXCR1 in different breast tissues and the relevance between its expression and efficacy of neo-adjuvant chemotherapy in breast cancer. Oncotarget.

[102] Ruffini PA (2019). The CXCL8-CXCR1/2 axis as a Therapeutic target in breast cancer stem-like cells. Front Oncol.

[103] Murugan AK, Grieco M, Tsuchida N (2019). RAS mutations in human cancers: Roles in precision medicine. Semin Cancer Biol.

[104] Kufareva I, Salanga CL, Handel TM (2015). Chemokine and chemokine receptor structure and interactions: Implications for therapeutic strategies. Immunol Cell Biol.

[105] Ning Y, Labonte MJ, Zhang W, Bohanes PO, Gerger A, Yang D, Benhaim L, Paez D, Rosenberg DO, Nagulapalli Venkata KC (2012). The CXCR2 antagonist, SCH-527123, shows antitumor activity and sensitizes cells to oxaliplatin in preclinical colon cancer models. Mol Cancer Ther.

[106] Singh S, Sadanandam A, Nannuru KC, Varney ML, Mayer-Ezell R, Bond R, Singh RK (2009). Small-molecule antagonists for CXCR2 and CXCR1 inhibit human melanoma growth by decreasing tumor cell proliferation, survival, and angiogenesis. Clin Cancer Res.

[107] Liu X, Peng J, Sun W, Yang S, Deng G, Li F, Cheng JW, Gordon JR (2012). G31P, an antagonist against CXC chemokine receptors 1 and 2, inhibits growth of human prostate cancer cells in nude mice. Tohoku J Exp Med.

[108] Bieche I, Chavey C, Andrieu C, Busson M, Vacher S, Le Corre L, Guinebretière JM, Burlinchon S, Lidereau R, Lazennec G (2007). CXC chemokines located in the 4q21 region are up-regulated in breast cancer. Endocr Relat Cancer.

[109] Varney ML, Singh S, Li A, Mayer-Ezell R, Bond R, Singh RK (2011). Small molecule antagonists for CXCR2 and CXCR1 inhibit human colon cancer liver metastases. Cancer Lett.

[110] Brandolini L, Cristiano L, Fidoamore A, De Pizzol M, Di Giacomo E, Florio TM, Confalone G, Galante A, Cinque B, Benedetti E (2015). Targeting CXCR1 on breast cancer stem cells: Signaling pathways and clinical application modelling. Oncotarget.

[111] Steele CW, Karim SA, Leach JDG, Bailey P, Upstill-Goddard R, Rishi L, Foth M, Bryson S, McDaid K, Wilson Z (2016). CXCR2 inhibition profoundly suppresses metastases and augments immunotherapy in pancreatic ductal adenocarcinoma. Cancer Cell.

[112] Hirose K, Hakozaki M, Nyunoya Y, Kobayashi Y, Matsushita K, Takenouchi T, Mikata A, Mukaida N, Matsushima K (1995). Chemokine gene transfection into tumour cells reduced tumorigenicity in nude mice in association with neutrophilic infiltration. Br J Cancer.

[113] Nair P, Gaga M, Zervas E, Alagha K, Hargreave FE, O'Byrne PM, Stryszak P, Gann L, Sadeh J, Chanez P, Study Investigators (2012). Safety and efficacy of a CXCR2 antagonist in patients with severe asthma and sputum neutrophils: A randomized, placebo-controlled clinical trial. Clin Exp Allergy.

[114] Allegretti M, Cesta MC, Garin A, Proudfoot AE (2012). Current status of chemokine receptor inhibitors in development. Immunol Lett.

[115] Maroulakou IG, Oemler W, Naber SP, Tsichlis PN (2007). Akt1 ablation inhibits, whereas Akt2 ablation accelerates, the development of mammary adenocarcinomas in mouse mammary tumor virus (MMTV)-ErbB2/neu and MMTV-polyoma middle T transgenic mice. Cancer Res.

[116] Liu H, Radisky DC, Nelson CM, Zhang H, Fata JE, Roth RA, Bissell MJ (2006). Mechanism of Akt1 inhibition of breast cancer cell invasion reveals a protumorigenic role for TSC2. Proc Natl Acad Sci USA.

[117] Li B, Hou D, Guo H, Zhou H, Zhang S, Xu X, Liu Q, Zhang X, Zou Y, Gong Y, Shao C (2017). Resveratrol sequentially induces replication and oxidative stresses to drive p53-CXCR2 mediated cellular senescence in cancer cells. Sci Rep.

